# Intraductal papilloma of minor salivary gland: a case report and literature review

**DOI:** 10.3389/froh.2025.1508614

**Published:** 2025-02-12

**Authors:** Yifei Wang, Xiaowei Li, Chunfang Yao, Yi Wu, Hengli Ni

**Affiliations:** Department of Pathology, Children's Hospital of Soochow University, Soochow University, Suzhou, Jiangsu, China

**Keywords:** intraductal papilloma, inverted ductal papilloma, ductal papilloma, cystadenoma, minor salivary gland

## Abstract

Intraductal papilloma is a rare benign neoplasm arising from the secretory ducts of the salivary glands. A 79-year-old man with an intraductal papilloma located in the minor salivary gland of sublingual mucosa was reported. The lesion, characterized by extensive internal papillomatous hyperplasia, was confirmed through histopathology and immunohistochemistry. We conducted a literature review to clarify intraductal papilloma and differences between inverted ductal papilloma, highlighting the significance of CK7 and MUC-1 immunohistochemical staining in diagnosing intraductal papilloma.

## Introduction

Intraductal papilloma (IP) is a rare small salivary gland tumor. In the 2017 and 2022 editions of the WHO classifications, it is classified with inverted ductal papilloma (IDP) as ductal papilloma of the oral cavity (DPOC) (1, 2). In published articles, case reports with a diagnosis of DPOC do not exceed 10 cases, and precise demographic information is not yet available. IP occurs in adults as a rare lesion of the parotid or sublingual gland in patients 60–75 years of age or older, with no gender preference, and is clinically characterized by a painless lesion under the oral mucosa (3, 4). IP and IDP consist of adenoepithelial or squamous epithelium that grows in patches in the submucosa, some of which are accompanied by mucus secretion, mainly involving the lower lip, sublingual oral mucosa and minor salivary glands (5). There are many histomorphologic similarities but no characteristic molecular alterations, and immunohistochemistry (IHC) is predominantly CK-positive, indicating epithelial origin. In this study, we report an intraductal papilloma located in the sublingual mucosa that was diagnosed by frozen section, formalin-fixed paraffin-embedding (FFPE) and IHC. Although intraductal papilloma is a benign tumor, the cellular morphology differs from that of IDP, and its relationship to salivary gland intraductal papillary mucinous neoplasm (SG-IPMN) remains unclear (6, 7). In this case, we analyzed the expression of tumor cells by various IHC methods and special stains, with a review of the literature, the cytokeratin 7 (CK7) and mucin 1 (MUC-1) expression patterns of IP tumor cells contribute to pathological diagnosis. This will add to the clinical experience with IP of minor salivary gland tumors.

## Case report

**Figure 1:**
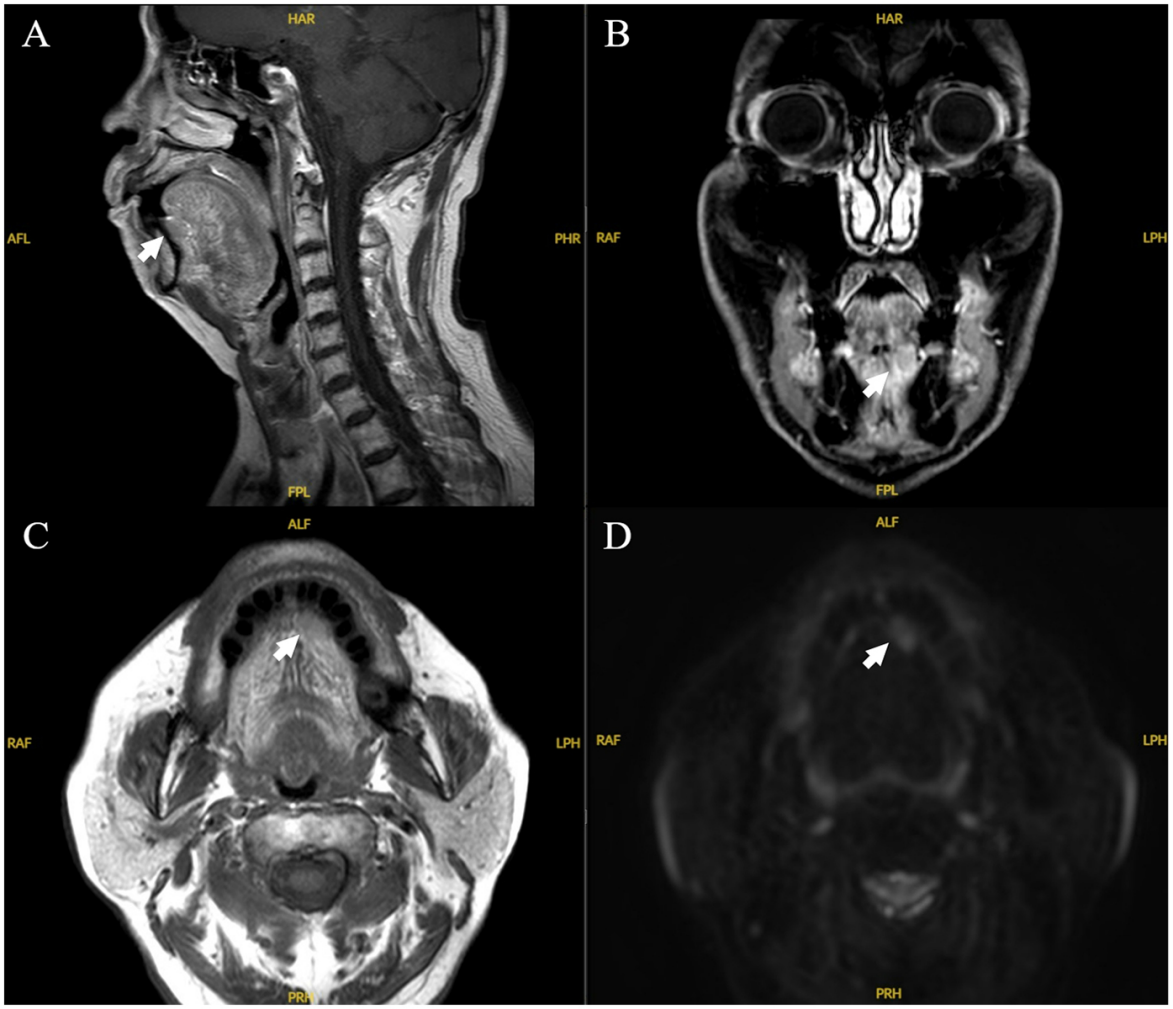
MRI imaging of the case. Sagittal **(A)**, coronal **(B)**, axial **(C)**, and axial diffusion weighted imaging **(D)** MRI images. There was a 2.0 cm × 1.8 cm hyperintense unicystic, regular mass with clear margins, located under left tongue.

A 79-year-old Chinese man presented with a 4-day history of a painless mass on the left sublingual mucosa in 2023. He had a 20-year history of hypertension and 15-year history of type 2 diabetes, which were currently being treated. In addition, the patient received a cardiac stent in 2020 and was on anti-thrombotic therapy (Table 1). He had no history of smoking and drinking, did not chew betel quid, had no history of localized trauma or surgery, and had no obvious trigger at this onset. On examination, a floret like mass 2 × 1.8 × 1.5 cm size, located on the mucosal surface beneath the tongue with slightly stiff borders, indistinct tenderness and no bleeding. The rest of the orofacial structures (teeth, lip, gums and mandible) were normal. CT and MRI-scan diagnosis was that of possible of tongue cancer (Figure 1), sublingual region and bilateral cervical zone I lymph nodes visible, did not exclude the possibility of metastasis.

**Table 1:** Medication use for the patient's comorbidities.

No.	Medication	Dosage	Frequency	Uses	Duration
1	Nifedipine sustained release	10 mg	QD	Hypertension	20 Y
2	Simvastatin	10 mg	QD	Hypertension	20 Y
3	Furosemide	20 mg	QD	Hypertension	18 Y
4	Gliclazide	30 mg	BID	Hypertension	18 Y
5	Metoprolol	30 mg	BID	Hypertension	20 Y
6	Spironolactone	20 mg	QD	Heart failure	15 Y
7	Metformin	0.25 g	BID	Type 2 diabetes	15 Y
8	Aspirin	1 tablet (75 mg)	QD	Coronary stents	4 Y
9	Poliviv	1 tablet (75 mg)	QD	Coronary stents	4 Y

BID, Bis in Die; QD, Quaque Die; Y, Year. The patient had long-standing hypertension and diabetes mellitus, and a coronary stent had been placed. Table 1 lists the associated medication used.

Although our patient had a history of hypertension, coronary artery disease, and type 2 diabetes, these were not considered to be the direct cause of the sublingual mucosal lesions. The mass was suspected to be carcinoma of the tongue and an extensive resection of the tumor was performed (oral surgery department). Squamous cell carcinoma (SCC) of the sublingual mucosa was highly suspected on the basis of lesion location, age, MRI-scan imaging and clinical features. However, the differential diagnosis also includes other diseases such as salivary duct cyst, mucoepidermoid carcinoma, acinar cell carcinoma, canalicular adenoma, and cystadenocarcinoma.

**Figure 2:**
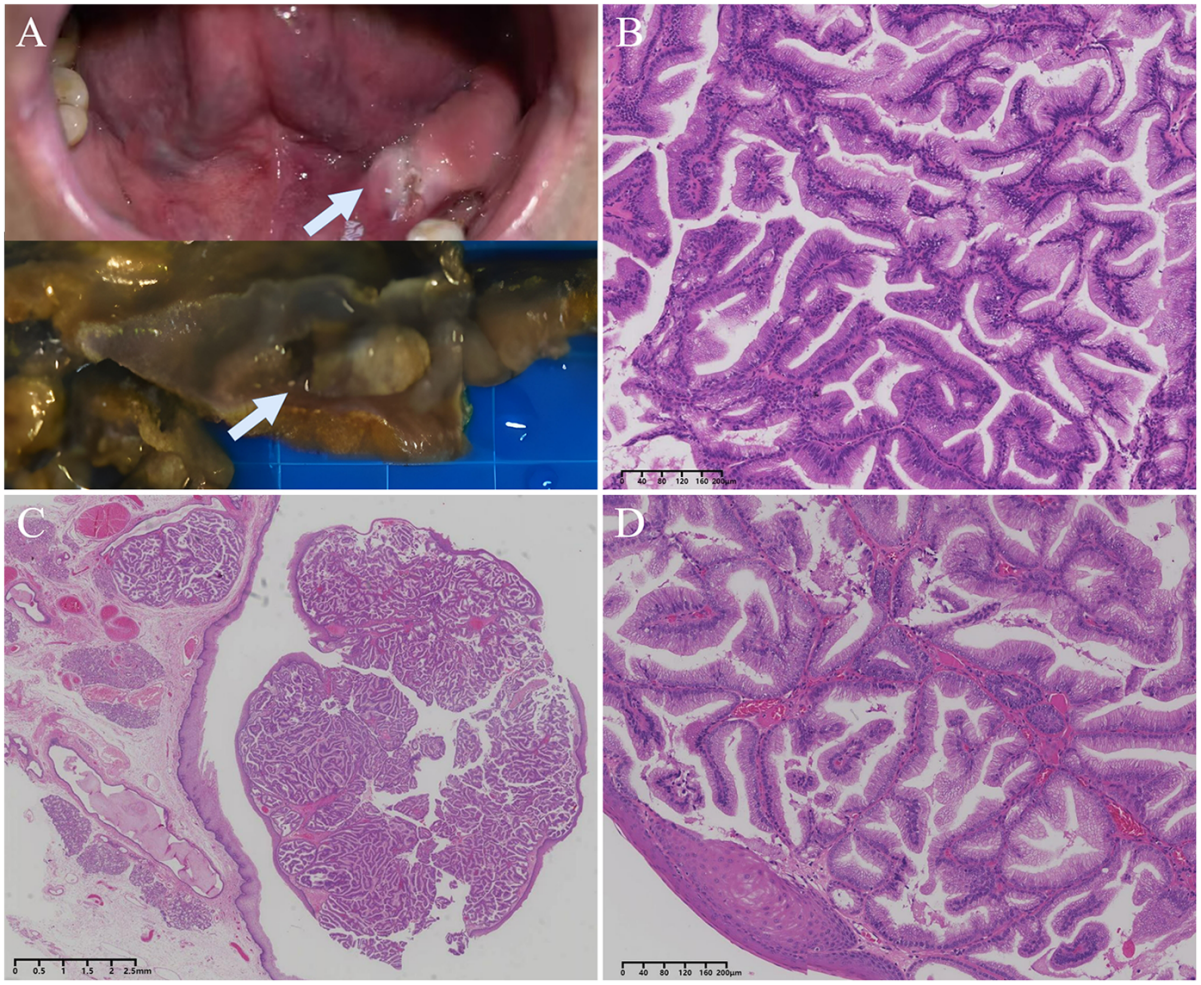
The gross appearance and histology of intraductal papilloma. **(A)** Clinical photograph (top) and surgical specimen (bottom) of the lesion, which was grey-white in colour, protruding from the mucosal surface and well demarcated from the surrounding tissue. **(B)** Frozen section showed papillary hyperplasia with densely arranged glandular epithelium, mild cells, and clear tumor boundary. **(C)** A low-power view of the FFPE section showed a well-defined submucosal nodular lesion consisting of papillary hyperplasia filled with cysts. **(D)** Intraluminal papillary protrusions of columnar cells with thin fibrovascular cores and containing mucus.

Peroperatively, a frozen-section examination was performed in order to decide orient the subsequent procedure. On gross examination, cauliflower like and elevated growth mass, centrally located in irregular mucosa, and its cut surface was off-white and taupe with slightly hard consistency material (Figure 2A). At frozen-section microscopy, the mass was well defined and located below the growth of squamous epithelium covering the oral mucosa, the mass consisted of densely proliferating high columnar glandular epithelium with well-defined ductal structure, well-differentiated columnar cells, the nuclei were neatly arranged, the nucleocytoplasmic ratio was similar to that of ductal cells (Figure 2B). This morphology is rare in oral surgery, and the morphology can rule out squamous cell carcinomas, the frozen-section diagnosis was that of possible adenomas or possible well-differentiated adenocarcinoma. Since the possibility of malignancy could not be excluded, the surgical procedure of “extended resection of the tumor on the floor of the mouth and dissection of the left neck lymph nodes” was continued.

Considering the history of coronary heart disease, class II cardiac function, combined with hypertension and diabetes mellitus, high surgical risk, large intraoperative blood pressure fluctuation, high blood loss, and risk of cardiogenic shock, the patient was transferred to Intensive care unit (ICU) for monitoring and treatment. 3 days after the surgical resection, the patient had an episode of atrial fibrillation, and the heart rhythm was controlled by cedi-lanid and cordarone, which was later converted to sinus rhythm. Blood sugar and blood pressure were controlled by insulin pump and urapidil hydrochloride. 7 days after the surgical resection, the patient was assessed for good swallowing and expectoration. The ventilator-assisted breathing was discontinued and replaced with artificial nasal breathing. 14 days after the surgical resection, the patient's general condition was good, and the vital signs were stable. The postoperative course was unevetful and no signs of recurrence or other salivary gland-related malignancies during 36 months clinical follow-up that included CT-scan examination.

## Histological study

On hematoxylin-eosin staining (H&E) sections, a dense proliferation of glandular patterns was deteted in the submucosal tissue composed of benign squamous epithelium. The tumor cells grew in papillary or glandular-tubular form, fibrous vascular axis can be seen in the center of papillary. The cells are mucinous columnar epithelium, arranged in monolayer or pseudostratified, the nucleus was located at the base, no keratinization or atypia, the mitotic figures are rare. The peri-lesion tissue showed multiple dilated ducts, and the glandular epithelium was accompanied by acidophilic metaplasia. (Figure 2C,D) The tumor tissue was completely embedded in frozen or FFPE, respectively, and no clear vascular invasion was detected. No tumor metastasis was seen in any of the lymph nodes sent for examination, and some lymph nodes showed only chronic inflammatory reactive enlargement. Although the tumor was in close proximity to the mucosal surface, no opening of the lesion into the surface epithelial cells was found in multiple sections.

**Figure 3:**
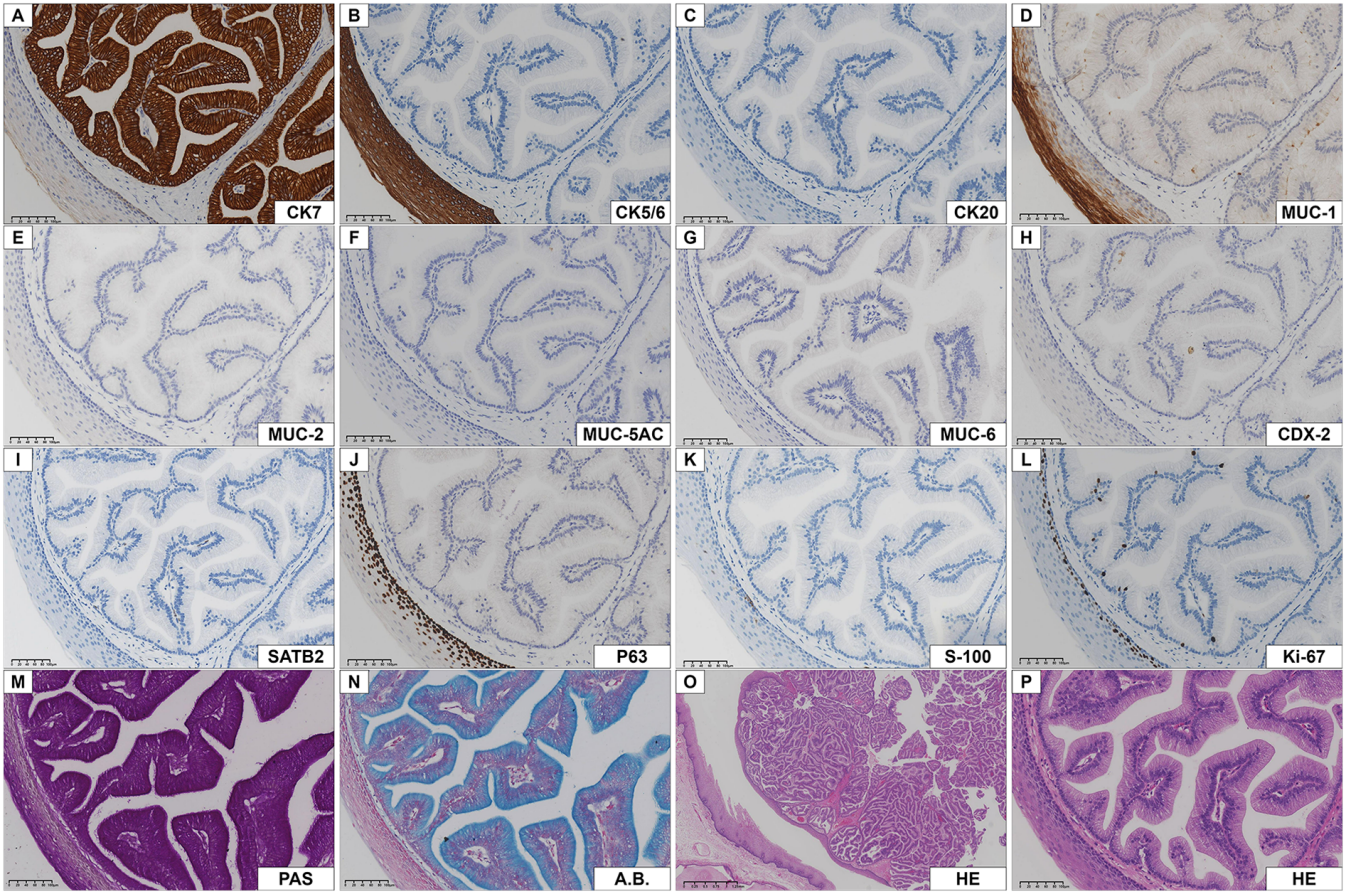
Immunohistochemistry of intraductal papilloma. **(A)** CK7 was strongly positive in columnar epithelial cells. (B, C) CK5/6 and CK20 were negative in tumor cells, CK5/6 was positive in the epithelium covering the tumor. **(D)** MUC-1 was positive in most tumor cells and duct cells. (E-I) MUC-2, MUC-5AC, MUC-6, CDX-2, SATB2 were negative in tumor cells. (J, K) P63 and S-100 were only positive in basal cells or myoepithelial cells. **(L)** The number of Ki-67 nuclear positive cells was low. (M, N) The neoplastic epithelium showed columnar epithelial cells cytoplasm with PAS and A.B. reactivity. (O, P) Low- and high-power HE staining of the same field of view showed a mass composed of benign adenoepithelium located beneath the squamous epithelium.

Squamous cell carcinoma could be initially excluded morphologically, but mucoepidermoid carcinoma, acinic cell carcinoma, ductal adenoma, cystadenocarcinoma, cystadenoma, intercalated duct adenoma, and metastatic tumors of the gastrointestinal tract could not be completely ruled out. To confirm the diagnosis and differential diagnosis, immunohistochemistry and special staining were subsequently performed. By IHC stains, the papillae were composed of mucous columnar cells, which expressed CK7 and did not express cytokeratin 20 (CK20). MUC-1 was focally expressed (approximatively in 50% of the cells), but not mucin 2 (MUC-2), mucin 5AC (MUC-5AC), and mucin 6 (MUC-6). Cytokeratin 5/6 (CK5/6) and P63 were only expressed in the tumor surface-coated squamous epithelium and in the myoepithelial cells of the glandular lumen, and S-100 protein (S-100) was only positive in myoepithelial cells. Very few cells showed Ki-67 (Mib-1) positive nuclei (approximatively 3%). The tumor cells were negative for special AT-rich sequence-binding protein 2 (SATB2) and caudal type homeobox 2 (CDX-2), which rules out the possibility that the tumor cells originate from the digestive tract (stomach, small intestine, colon, etc.). By special chemical stains, cytoplasmic granules were positive for periodic acid-schiff (PAS) and algal blue (A.B.) staining (Figure 3).

Pathohistologically, the tumor grew in a slightly circular pattern, protruding from the mucosal surface with well-defined borders. The lesion was mainly located between the minor salivary gland secretory ducts and the oral surface epithelium. The tumor consists of glandular epithelial cells without cytological atypia, covered with normal squamous epithelium. Such highly columnar cells, expressing CK7 and MUC-1, PAS and A.B. showed cellular mucus and low expression of Ki-67. Based on the above findings, the diagnosis was that of intraductal papilloma.

## Molecular pathology

For further diagnosis, we performed fluorescence *in situ* hybridization staining (FISH) of FFPE specimens for the *MAML2* (11q21) gene break and the *ETV6/NTRK3* gene fusion. Most mucoepidermoid carcinoma will have a *CRTC1-MAML2* or *CRTC3-MAML2* fusion, and *ETV6/NTRK3* is a common measured molecular alteration in secretory carcinomas (8, 9). However, in the present case, neither *MAML2* gene breaks nor *ETV6/NTRK3* fusion genes were detected in the tumor by FISH.

## Differential diagnosis

Differential diagnosis was performed during pathological diagnosis. By H&E morphology, no obvious squamous cell differentiation was found, nuclear mitosis was rare, and the border was clear, which was not consistent with squamous cell carcinoma. Although S-100 was partially positive and had intracellular mucus, the cells were not eosinophilic, and there were no squamous epidermoid, and intermediate cells, so mucoepidermoid carcinoma was not considered. Acinic cell carcinoma (AciCC) is characterized by plasmacytoid salivary vesicles, which are composed of a variety of cells that are slightly basophilic and form small glandular luminal structures; in this case, the tumor did not have vacuolated or microcystic structures, and PAS positivity could rule out the possibility of AciCC. Canalicular adenoma is composed of high columnar cells, which form a bead-like structure, and the mesenchyme is lax and rich in blood vessels, but this feature was not present in this case. Cystadenocarcinoma and cystadenoma were characterized by multiple cystic lumens with fibrovascular axons, but this case had a single cystic lumen, and the P63-positive myoepithelial cells were only focally distributed. The morphology of intercalated duct adenoma was also very similar to the present case, but the intercalated duct adenoma has P63 and S100 positive myoepithelial cells, which was the biggest point of differentiation from this case. The Ki-67 in this case was about 3%, the tumor border was clear, and no tumor lymph node metastasis was seen, which was not consistent with a highly malignant tumor. Some of the cells were morphologically similar to intestinal villous adenoma, but they were negative by CK20, SATB2, and CDX-2, so ectopic or tumor metastasis in the digestive mucosa was excluded.

## Discussion

Ductal papilloma of the oral cavity (DPOC) is benign papillary tumor consisting mainly of intraductal papilloma (IP) and inverted ductal papilloma (IDP) by the 2017 and 2022 WHO classifications (4, 10). Intraductal papilloma is a rare disease, which is most common in the major duct of minor salivary glands (11, 12). Intraductal papilloma cause cystic formation and are usually too small to detect by conventional techniques (13). Precise diagnosis of this tumor before surgery is difficult. Ultrasound is of little diagnostic value, and CT and MRI scans may be helpful in assessing the location and extent of lesions (14). Fine-needle aspiration (FNA) appears to be the best method for preoperative diagnosis of parotid gland tumors, but DPOC is not suitable for FNA, and biopsy is more suitable for diagnosis (12, 15).

Histologically, inverted ductal papilloma is described as a papillary structure composed of basal or epithelioid cells with uniform morphology (16). However, the papillary structure of intraductal papilloma is more composed of glandular epithelium, goblet cells or columnar cuboid epithelium, neatly arranged, intracellular secretions can be seen, and the cell cytoplasm is abundant. Few myoepithelial or basal cells were detected in the papillary structures of the tumor. DPOC are uniluminal cystic lesions with papillary growth of flat, columnar cells in the lumen, mucinous cells scattered or clustered in the DPOC, cells lacking mitosis and atypia, and no invasive growth.

Although *PIK3CA* or *AKT* mutations are more common in breast intraductal papilloma, *AKT1 E17 K* mutation was detected in intraductal papilloma of minor salivary glands, and no genetic alterations (*BRAF*, *PI3KCA* etc.) were detected in salivary glands intraductal papilloma with previous reports (6, 17, 18). Amplitude-based massively parallel processor sequencing revealed that all 3 patients had the same AKT1 mutation, but no co-occurring mutational pathways in other genes of *RAS* or *PI3 K*. Although the detection of human papillomavirus (HPV) subtypes 6/11 in IDP cases has been reported, it does not indicate that there is a correlation between IDP and HPV infection, no HPV DNA cases have been detected in intraductal papilloma, and no cytopathological changes suggestive of viral infection have been observed in our case (19).

From 2000 to 2022, 15 new cases of intraductal papilloma have been reported (3, 18, 20–24), including the present case (Table 2). In this literature review, patients with lesions ranged in age from 8 to 79 years, usually between 60 and 75 years, with a mean age of 60 years, and were predominantly of male gender (9/15). Intraductal papilloma are most often located in the mucosa under the tongue and appear nodular. The outlet of the salivary gland secretory duct is usually dilated, and the lesion is usually inconspicuous with the mucosal surface. There may be separate bands of fibrous connective tissue, and cystic dilatation can be seen in the tumor. In immunohistochemistry, CK7 (4/4) and MUC-1 (5/5) were positive in tumor cells, and P63 and S-100 proteins were only positive in myoepithelial cells. Given the low-grade cytology and low mitotic profile of the tumor, the prognosis is excellent, the risk of recurrence is low, metastases are rare in only a few cases, and there is essentially no disease-related mortality.

**Table 2:** Reported cases of intraductal papilloma and their clinical features.

No. cases	Diagnosis	Sex/age	Size (cm)	Location	CK7	MUC-1	P63	S100	References
1	IP	F/39	1.5	Oral floor	ND	ND	ND	ND	Masato Nakaguro et al. (6)
2	IP	F/46	1.0	Buccal mucosa	ND	ND	ND	ND	Masato Nakaguro et al. (7)
3	IPMN	F/78	2.0	Minor salivary gland	ND	ND	–	–	Abbas Agaimy et al. (17)
4	IPMN	M/71	1.5	Minor salivary gland	ND	+	Single basal cells+	–	Abbas Agaimy et al. (17)
5	IPMN	M/65	2.5	Palate arch	ND	+	–	–	Abbas Agaimy et al. (17)
6	IP	M/70	2.0	Oral floor	ND	ND	ND	+	Toshitaka Nagao et al. (18)
7	IP with invasive	M/72	2.0	Cervical region	ND	ND	ND	+	Toshitaka Nagao et al. (18)
8	IP	M/77	1.1	Oral floor	ND	ND	ND	ND	Agnes Assao et al. (3)
9	IP	M/76	1.0	Left submandibular salivary gland	ND	ND	ND	ND	Showkat Mirza et al. (20)
10	IP	F/8	2.0	Right parotid gland	ND	ND	ND	ND	Hulya Noseri et al. (15)
11	IP	F/71	0.8	Left lower vestibular area	ND	ND	ND	ND	Yuk Kwan Chen et al. (21)
12	IP	M/47	0.6	Sublingual mucosa	+	ND	–	ND	Aikawa Tomonao et al. (22)
13	IP	F/30	1.7	Minor salivary glands of the larynx	+	+	–	–	Satoshi Hara et al. (24)
14	IP	M/60	2.0	Right buccal mucosa	+	+	Single basal cells+	ND	Eleni Marina Kalogirou et al. (23)
15	IP	M/79	2.0	Sublingual mucosa	+	+	–	–	Present case

F, female; M, male; NA, not available; +, indicates positive; −, negative; ND, not done because of tissue limitation; IP, intraductal papilloma; IPMN, intraductal papillary mucinous neoplasm. Immunohistochemical CK7, MUC-1, P63, S-100 expression and clinicopathologic features of new cases of oral intraductal papillomas published since 2001 until the present case.

In the past, salivary gland papilloma (SP), intraductal papilloma, cystadenoma, and cystadenocarcinoma were classified as minor salivary gland papillary lesions. In recent years, some scholars have proposed the concept of SG-IPMN, as in the pancreas, epithelial cells can develop atypical, carcinoma *in situ*, or invasive growth. With the development of sanger sequencing, mutations in *AKT1 E17 K* and *HRAS Q61R* were found in minor salivary SG-IPMN (6, 17). However, the pathological diagnosis of these diseases is somewhat confusing and reproducible, and there are differences in conceptual understanding between oral surgeon and pathologists.

In our case, a well-circumscribed papillary growth mass with mild cells, but inconspicuous cystic dilatation and lack of molecular alterations made the diagnosis of IPMN of the minor salivary gland less evidence-based, and the final diagnosis of intraductal papilloma was more reasonable. CK7 and MUC-1 immunohistochemical staining has certain guiding value in the diagnosis of intraductal papilloma.

We also advocate the need to subdivide DPOC according to epithelial type, with inverted ductal papilloma being squamous epithelium and intraductal papilloma being more of an adenoidal epithelium. Site and differentiation lead to different mucus composition within the glandular epithelium, causing mucus accumulation within the mass, CK7 and MUC-1 can be used to classify epithelial origin. Since papillary lesions including intraductal papilloma are rare, more clinical data, especially molecular pathological changes, are needed to provide evidence for clinical treatment and pathological diagnosis.

## Data Availability

The original contributions presented in the study are included in the article/Supplementary Material, further inquiries can be directed to the corresponding author.
